# Reimagining the Administrative State in Times of Global Health Crisis: An Anatomy of Taiwan’s Regulatory Actions in Response to the COVID-19 Pandemic

**DOI:** 10.1017/err.2020.25

**Published:** 2020-04-02

**Authors:** Ching-Fu LIN, Chien-Huei WU, Chuan-Feng WU

**Affiliations:** * Associate Professor, Institute of Law for Science and Technology, National Tsing Hua University, Taiwan; email: chingfulin@mx.nthu.edu.tw.; ** Associate Research Fellow, Institute of European and American Studies, Academia Sinica, Taiwan.; *** Associate Research Fellow, Institutum Iurisprudentiae, Academia Sinica, Taiwan.

## I. Introduction

Taiwan is standing in the midst of the global novel coronavirus (COVID-19) crisis. At the time of this writing, in Taiwan, 267 people (227 imported and 40 indigenous cases) are infected with this severe acute respiratory syndrome and 2 people have died.^1^ The entire country is bracing not only for the public health challenges posed by the outbreak that respects no boundaries, but also for the social, economic and political ramifications.

The regulation and governance of communicable diseases in Taiwan are underpinned by the Communicable Disease Control Act (CDC Act).^2^ The law serves as a framework for government actions during public health emergencies, including *inter alia* setting up a disease control network by dividing the country into regions^3^; establishing a centralised platform to command and coordinate agencies’ actions, share information and respond to inquiries^4^; integrating personnel, facilities and resources in preparation for outbreaks^5^; issuing voluntary and mandatory isolation orders^6^; and implementing border restrictions.^7^ A closer look at the legislation as well as more recent developments suggests an explicit institutional design choice that upholds technocracy and delegates significant authority and broad discretion to health experts in the Executive Branch to steer the country in times of emergency, entrenching an administrative state^8^ in this young democracy.

In January of this year, when China decided to lock down Wuhan due to COVID-19, Taiwan took an exceptionally precautionary approach based on three major factors^9^: Taiwan’s decades-long exclusion from the international health community without World Health Organization (WHO) membership/observership and access to other arms of the United Nations (UN)^10^; traumatic experiences during the 2003 severe acute respiratory syndrome (SARS) outbreak, where regulatory failure led to a wave of reforms^11^; and the island’s geographical proximity to and geopolitical suspicion of China. Because international exclusion has prevented Taiwan from benefitting through information-sharing and collective efforts underpinned by the multilateral WHO framework,^12^ the country has developed a “self-help” approach in countering possible public health threats, which has partly resulted in a heavy reliance on technocratic decision-making.^13^ This sense of exclusion and the need for “self-help” was strengthened by bitter SARS experiences, when the WHO offered limited help.^14^ During the SARS outbreak, Taiwan lost 73 lives, had hundreds of its citizens hospitalised and experienced the controversial lockdown of Ho-Ping Hospital in Taipei,^15^ and such a history has prompted Taiwan to take a highly precautionary approach. Furthermore, COVID-19 and SARS share the same origin – China – and everything related to China seems controversial in Taiwan (even more so after the current administration took power in 2016) because of the complex historical, social, economic and political relationship between the two countries. China earned a track record for concealing information during the SARS outbreak,^16^ and it is widely believed in Taiwan that China has done the same regarding COVID-19. Economically, Taiwanese enterprises invest intensively in Wuhan and other provinces in China, which results in frequent travel between the two countries. Socially, a number of Taiwanese marry Chinese citizens, and the Lunar New Year is a time for family reunions, which may have contributed to virus transmission from China to Taiwan. Finally, and most importantly, the current administration in Taiwan is suspicious of China, which leads it to take a sober look at the information and news released by China and to take a precautionary approach in countering COVID-19.

However, Taiwan’s proactive and precautionary response to COVID-19 might at the same time pose a threat to this young democracy. The critical issue here is how a constitutional democracy survives a public health emergency with the unprecedented magnitude of COVID-19. To be sure, governments worldwide have adopted a wide range of regulatory measures,^17^ such as social distancing and stay-home orders,^18^ mass testing,^19^ contact-tracing and surveillance,^20^ the shuttering of non-essential services^21^ and even border closures and national shutdowns.^22^ The legality and legitimacy of such regulatory actions may be examined pursuant to different norms and standards underlying a country’s respective political system, legal tradition and social context. On the one hand, an emergency calls for stronger and swifter governmental actions to safeguard public health and national security in a manner that may not allow sufficient time for parliamentary deliberation or traditional administrative procedures.^23^ The urgency to take effective and efficient measures oftentimes justifies a wide margin of executive discretion. On the other hand, measures adopted in times of emergency tend to linger and become normalised. Emergencies frequently serve to expand the power of executive agencies and side-line legislative or even judicial gatekeepers or lead to irreversible harm to fundamental human rights.^24^ In Taiwan, among those measures adopted to combat COVID-19, the Special Act for Prevention, Relief and Revitalization Measures for Severe Pneumonia with Novel Pathogens (COVID-19 Special Act) seems to have triggered controversial questions about the bending and even breaking of fundamental human rights and core legal principles. This paper therefore aims to offer an anatomy of Taiwan’s regulatory actions taken in response to the global COVID-19 pandemic, assess their implications for risk regulation and governance in a global context and urge a reimagining of the administrative state in the – hopefully – post-COVID-19 world.

## II. Taiwan’s regulatory actions: an anatomy

Public health emergencies such as COVID-19 usually compel governments to steer, coordinate and respond with various measures. Taiwan’s CDC Act, which was carefully re-evaluated and amended after the SARS outbreak, serves as the primary legal basis.^25^ Overall, executive agencies are granted considerable power to adopt various regulatory measures to cope with imminent epidemics and public health threats, and such agencies enjoy a substantial margin of discretion under urgent circumstances. Yet the government has so far treated the COVID-19 outbreak as a national security issue and has adopted extraordinary interventions reflecting such a framing.^26^ While these interventions have been largely effective and have garnered majority support from society, they might also risk breaking fundamental rule of law principles, causing irreversible harms to human rights and bending the country’s constitutional order with lasting and systematic effects.^27^ Here, we examine some key measures taken by the government in Taiwan along three essential dimensions of risk regulation, providing an anatomy of Taiwan’s actions in its whole-of-society and global approach.

### 1. Advanced but informed risk assessment?

To address a rapidly changing pandemic of global concern that is uncertain and dynamic in nature, the right timing for regulatory action is always a guesstimate and can only be evaluated in hindsight. Acting too early and taking excessive precautions risk being criticised *ex post* as paranoid and unduly disrupting the market and society; acting too late may cause irreversible damage and miss the window of opportunity to tackle the problem. Therefore, the key to successful pandemic response is to determine how and when a specific event becomes a public health emergency with limited scientific data.^28^ This is by no means an easy task.

Taiwan has learned from its SARS experiences that timely and accurate determinations about a public health emergency may pose a formidable challenge if the roles and responsibilities in the decision-making process are not clearly defined (eg tensions and overlapping jurisdictions between local and central governments) and if scientific evidence is inadequate, complicated or unavailable (eg exclusion from the WHO and failure to receive critical information).^29^ To establish a unified authority and decision-making process,^30^ Article 8 of the CDC Act grants the Ministry of Health and Welfare, particularly the Centers for Disease Control, the exclusive power and responsibility to determine public health emergencies and announce them with a single voice. This further entrenches the technocratic anchor in Taiwan’s legal framework for communicable disease control, which relies heavily on the medical profession, as it has long enjoyed prestigious status in society and has generally been regarded as a trusted expert voice in policy-making.

When the world saw only uncertain signs of mysterious pneumonia cases in Wuhan in December 2019, Taiwan treated them with the utmost urgency.^31^ On 31 December 2019, when China silenced doctors from disclosing information, the Taiwanese government warned China and the WHO International Health Regulations (IHR) Contact Points of the danger of human-to-human transmission^32^ and began to send officials to board all direct flights from Wuhan and inspect passengers for fever or pneumonia symptoms, prepared contact tracking and tracing mechanisms and surveyed the availability of medical supplies.^33^ Regardless of the insufficiency of scientific evidence and clinical data at the time, the government spent great effort on assessing whether the disease constituted an emergency.^34^ Taiwan sent two experts to Wuhan to obtain more information on the outbreak.^35^ On 15 January 2020, Taiwan officially determined COVID-19 to be a Category V Communicable Disease,^36^ setting up the Central Epidemics Command Center (CECC) on 20 January to coordinate all control measures across various agencies.^37^ Following an advanced risk assessment, and recognising the need to act swiftly, the CECC moved to prevent Wuhan residents from entering the country on 23 January 2020, suspended tours to China on 25 January and, in the end, banned all Chinese visitors on 6 February.^38^

Yet, such an “advanced” risk assessment was not necessarily science-based or informed by sufficient clinical data, but was rather a multifaceted decision based on a complex weighing and balancing of technical, social, economic and political factors. First, because of China’s concealment of information in the early stages of the outbreak and the WHO’s failure to take appropriate action, there was very little scientific evidence or international guidance for risk assessment. Second, due to the tensions between Taiwan and China and the former’s exclusion from the WHO, the management of risks in public health emergencies is not merely a technical and scientific issue, but also a socioeconomic and even a political task. The government is forced to make decisions in the absence of solid data and to take additional steps ahead of emergencies by using its own judgement. Thus, public health risk assessment goes beyond disease control and extends to holistic social, economic and political management.^39^ This reflects a classic paradox in risk regulation^40^: if a country adopts a precautionary approach and engages in advanced preparedness, such a decision is thus to be made in the absence of sufficient data and scientific certainty and to run counter to the principle of scientific-based decision-making. Transparency and reasoning requirements are likely to be side-lined in the decision-making process. In times of emergency, such a paradox is exacerbated by the urgency and uncertainty of an event and may only be partly addressed by *ex post* review and accountability mechanisms, which are also prone to hindsight bias.

### 2. Effective and efficient risk communication

Risk communication plays an important role in tackling public health emergencies. Effective and efficient risk communication informs people of potential hazards and ensures public trust, which serves as the very premise of successful control measures. In order for complicated technical information to be conveyed to the lay public and subsequently transformed into concrete disease control measures, the Taiwanese government has employed both formal and informal channels (such as social media platforms and messengers) and has also worked with civic technology groups to facilitate two-way communication between the CECC and the public on a daily basis.^41^ Information about new imported and indigenous cases, global prevalence, new and revised control policies, clarification of misinformation and even the availability of medical masks and household necessities is provided at least once a day in a national press conference, followed by an open session for questions from the media and the public.^42^

The government has also taken measures to counter fake news, or disinformation. Taiwan has fought a long battle with fake news (China is the primary origin) since 2012 and has modernised its legislative and regulatory responses.^43^ Citing the CDC Act and the Social Order Maintenance Act (SOM Act), the government has fined or even arrested individuals for spreading rumours regarding COVID-19 and the shortage of household necessities.^44^ These measures may seem well-intentioned and make some sense in theory, but they may lack the scope of sensitivity regarding what is at stake. Some criticise the fairly broad authority and substantial discretion extended by law to competent authorities in determining when and what information should be contained and punished.^45^ If the government fails to strike a proper yet difficult balance,^46^ the fight against fake news and misinformation may ultimately backfire, resulting in a chilling effect that causes further information withholding, self-censorship and poor communication. Noting this dilemma, the government is gradually shifting to an open communication method that aims to actively inform the public via social media and rectify the marketplace of speech with correct information in a timely manner.

Overall, the CECC has played a successful role in establishing and maintaining a credible channel for communicating information to the public in plain language and in a timely and consistent manner. There is also a responsive mechanism to address public enquiries, concerns and criticisms, which promotes public awareness and ensures constructive dialogue without politicising key messages. This open, responsive and credible risk communication helps the government build public trust when imposing necessary restrictive measures, encouraging citizens to self-report their health status and debating risk management options and broader policies.

### 3. Proactive risk management

Taiwan has taken various proactive and swift risk management measures to control the spread of COVID-19.^47^ Here, we examine three dimensions of Taiwan’s risk management strategy: optimisation of key medical supplies; border control and travel bans; and big data-powered mechanisms for tracking and tracing the illness.

#### a. Optimisation of essential medical resources

The outbreak has led to a global shortage of medical masks. Taiwan first banned the export of medical masks on 24 January 2020, and further implemented a nationwide requisition and real-name rationing scheme premised upon the National Health Insurance (NHI) system after only a few days.^48^ From an economic perspective, the government has taken necessary measures to prioritise the acquisition and allocation of key medical supplies. To ensure a steady domestic supply, the government even drew up a budget of US$6.66 million to invest in equipment and 60 production lines by way of public–private partnerships.^49^ Taiwan’s supplies of medical masks have since risen at a steady pace, growing from daily manufacturing capability at 2.44 million units on 22 January to 13 million units in early March 2020.^50^ Such a surge in domestic medical mask production has made possible special allocations to medical personnel, airport control and schools. Taiwan further agreed to provide 100,000 masks per week to the USA in exchange for protective medical clothing when its production capacity is stabilised.^51^

In the process of optimising the supply of medical masks, however, a more ethics-sensitive framework to address distributional justice issues (eg unequal access to medical mask outlets in practice due to urban–rural differences) was overlooked. This can also be seen in the government’s management of medical resources in disaster preparedness planning. In Taiwan, the Communicable Disease Control Medical Network (CDCMN) was established following the SARS outbreak to centralise and optimise medical resources,^52^ under which designated infection hospitals would be appointed as centres for the provision of treatment and resource mobilisation during public health emergencies. The CDCMN also facilitated the expansion of negative-pressure isolation rooms (1100 beds)^53^ and isolation rooms (21,000 beds).^54^ Yet this system and its methodology for prioritisation are predominantly based on biomedical models and medical resources rationing. Accordingly, they may overlook complex socioeconomic dynamics and varying local needs.^55^ A framework for healthcare rationing and clinical prioritisation, especially under such exceptional circumstances, is desirable. Understandably, it may not be possible to set up *ex ante* criteria due to the uncertainty of an emergency. As such, extending broad discretion to physicians seems justifiable.^56^ Yet, even so, legal and ethical guidance that strikes a balance between efficiency and fairness,^57^ and transparency and due process in different circumstances, is much needed, as it can help guide the process of rationing medical resources^58^ and facilitate *ex post* review and accountability mechanisms.^59^

#### b. Border control and travel bans

Because cross-border travel constitutes the primary source of COVID-19 transmission for Taiwan, it proactively imposed entry restrictions for people from China as early as January 2020.^60^ Such restrictions were subsequently widened, and travellers from other countries listed on the Travel Notice were required to be quarantined for 14 days, eventually followed by a ban of all inbound foreign visitors on 9 March 2020.^61^ In addition to prohibiting inbound travel, outbound travel is also restricted: medical personnel, civil servants and middle- and primary-school teachers, students and staff are prohibited or strongly discouraged from travelling abroad, with a view towards maintaining human medical resources and avoiding transmission among vulnerable groups.^62^ These border control measures and travel bans are supported by the general public in the name of public interest at the expense of individual liberty.^63^

In times of health emergencies, public interest may serve as a justification for the government to bypass the legal imperatives and bring about a downwards cycle.^64^ An illustrative example is the CECC’s decision to publish the names of quarantined individuals who travel “unnecessarily” to these countries and to deprive their eligibility for the quarantine subsidy.^65^ Whereas the CDC Act, in Article 58(I)(4), authorises the government to impose home-based quarantine on individuals entering Taiwan from the worst-affected countries, the publication of the names of the quarantined individuals is without legal basis and disproportionately invades individuals’ privacy. The travel bans also suffer from the same weaknesses. Article 5(I) of the Immigration Act authorises the government to restrict the freedom to travel abroad for individuals involved in national security, yet neither medical professionals nor teachers/students are subject to the Act.^66^

#### c. Big data and surveillance

To have a clear picture of the travel and contact history, health records, real-time movement and other relevant information of (potentially) infected persons, the government has resorted to “big data” technologies to facilitate the effective implementation of quarantine measures and efficient contact tracing and locating. For instance, the government has actively integrated individuals’ travel records from the National Immigration Agency with other existing databases, such as the NHI and even police systems. Physicians then have a channel by which to access a patient’s travel history through the NHI card reader. The government also used the NHI database, which contains the entire population’s medical records, to review all patients’ health status and to locate suspected patients. Several patients’ daily routes, explored through surveillance cameras and mobile phone tracking, were disclosed to the public so that people who have visited these places can be on alert.^67^ A monitoring system, which can track individuals through GPS using their mobile devices and receive their daily health reports, was built to ensure individuals’ compliance with the quarantine order.^68^

Despite their effectiveness, these measures, with their great potential to infringe upon individuals’ privacy, were not carefully scrutinised according to rule of law and constitutional principles. Indeed, aggregating databases and mobility data can help fight COVID-19 by mapping travel and contact history, measuring social distancing and ensuring compliance with quarantine orders, but only when “appropriate legal, organizational, and computational safeguards [are] in place”.^69^ The Personal Data Protection Act sets out rules for collecting, processing and using personal data, such as lawfulness, purpose limitation, data minimisation and data security,^70^ but it has long been plagued by inflexible legal transplant and legal formalism without taking into account local contexts, and it has failed to provide a healthy regulatory environment. Furthermore, while the CDC Act^71^ and the COVID-19 Special Act^72^ authorise the government to impose quarantine, isolation care and “other necessary measures”, it remains debatable whether linking multiple national databases, disclosing names, collecting mobile data and analysing surveillance data qualify as “necessary measures” and pass the scrutiny of fundamental rule of law principles. Additionally, there is no notification process, revision mechanism or appeal process for those affected to challenge and seek dispute resolution.^73^ Again, these formal requirements merely serve as minimum safeguards and by no means suffice. A stronger mechanism that ensures deliberation, transparency and accountability by dynamic agency interaction and oversight is urgently needed.

In light of this development, the Taiwan Association for Human Rights (TAHR) has raised the question of whether privacy protection mechanisms, such as proportionality, transparency, due process and expiration, are overlooked in the process.^74^ In a crisis-driven fashion, mass surveillance techniques have become a convenient tool for the government to employ, with the aim of protecting people from COVID-19.^75^ The bottom line is fairly straightforward: while some exceptional measures may be justified by a “legality of emergency”,^76^ ignoring existing rule of law and human rights safeguards seems to be a dangerous move and may ultimately institutionalise exceptionalism and leave a lasting impact on the legal system and culture.^77^

## III. Contextualising Taiwan’s experience: a reimagining of the administrative state and global health governance

In combating COVID-19, the Executive Branch of government in Taiwan has enjoyed technocratic legitimacy and has played a dominant role. By enacting the COVID-19 Special Act in a swift manner, the Legislature further empowered the Executive Branch to exercise significant authority by way of broad and ill-defined delegation. While such “an open-ended grant of authority is in tension with the overriding aim of presenting the emergency regime as a temporary and limited exception to the principles of limited government”,^78^ are lawmakers aptly equipped to provide stronger and clearer guidance beyond delegation in times of public health emergencies? What are the implications of Taiwan’s experience for the administrative state and global health governance? Here, we further unpack and contextualise Taiwan’s experience in order to assess its broader, normative ramifications. As a developmental state, technocrats and experts tend to have an upper hand in shaping the direction and content of policies in Taiwan. In times of emergency, the need for a swift response favours the entrenchment of the administrative state at the expense of representative, democratic legitimacy. Furthermore, the nature of public health emergencies that respect no boundaries introduces a “double-jeopardy” for Taiwan’s Legislature to guide and oversee the Executive Branch. On the one hand, because Taiwan is excluded from the international health community, the Legislature is not equipped with international guidelines or best practices to “correct” domestic measures that may verge on arbitrariness or abuses. On the other hand, even in the context of global health governance, policies are primarily shaped by technocrats and experts, where the domestic legislature has limited access.^79^ The expansion of Executive power and technocracy appears unavoidable in an area of specialisation such as public health and in a time of emergency.^80^ Yet, even so, can we harness the administrative state with only traditional safeguards such as due process, proportionality and judicial review? What are the merits of a dynamic deliberation and accountability mechanism? Most importantly, how should we reconfigure the administrative state post-COVID-19?

Albeit marginalised, lawyers’ input into the debates and discourses in countering COVID-19 would seem ever more urgent. Haunted by the ghost of martial law, Taiwan strives to maintain a high degree of democratic governance and rule of law. This in part explains why the government has been reluctant to issue an across-the-board lockdown that bears an authoritarian legacy. The same also applies to the Legislature’s expeditious enactment of the COVID-19 Special Act as a legal basis for countering and containing the epidemic, with a view to fulfilling minimum democratic legitimacy and rule of law. Similarly, whereas the opposition party proposed that the President should invoke Article 43 of the Constitution to issue emergency decrees,^81^ the President has so far declined to do so on the practical ground of the sufficiency of the COVID-19 Special Act, and perhaps also on the ideological ground of past bitter experiences. Nonetheless, constitutional and legal guarantees of human rights and the rule of law extend far beyond a formal legal basis for emergency-response measures. In recognition of broad and unlimited delegation, as endorsed in Article 7 of the COVID-19 Special Act, some members of the Legislative Yuan (Legislature of Taiwan) have proposed an amendment with more stringent rules designed to strengthen democratic oversight, remedy rule of law deficits and prevent systematic failure.^82^ Relatedly, Taiwan’s dynamic and robust civil society serves as an additional safety net for extreme measures in the name of emergencies.^83^ In particular, the TAHR issued a statement emphasising the importance of democratic governance and the rule of law in times of pandemic, calling for resistance to blank-cheque authorisation.^84^ Yet sticking to such a formalist understanding of legal safeguards remains inadequate. A broader socio-political mechanism that involves different stakeholders to ensure constructive deliberation and to address challenges seems desirable.

Taiwan’s experience should also be read together with the dynamics of global health governance. Despite its alleged failure, the WHO continues to play a key role in providing guidance, expertise, technical assistance, a collaborative forum and personnel and practical tools, especially when the entire globe has been affected by COVID-19.^85^ From Taiwan’s perspective, meaningful participation in the WHO has been of the highest priority in its foreign relations, both for political reasons and for public health concerns. However, there is growing resentment of this organisation due to the sense of exclusion and the belief that is shared by at least some Taiwanese that this organisation uncritically takes positions that are overly favourable to China. Taiwan’s scepticism towards the WHO and its relationship with China has driven the country’s precautionary approach to coping with COVID-19. For the moment at least, the WHO seems to have lost its legitimacy and authority in global health governance and, subsequently, its charm for the Taiwanese people. Taiwan’s experience may further fuel the existing momentum in favour of a global call for WHO reforms.

The changing global order represents yet another context by which to expound upon Taiwan’s actions in fighting COVID-19 and its pursuit of health diplomacy. The growing influence of China in international organisations is due in part to the US’s turn from multilateralism to bilateralism, particularly under the Trump Administration.^86^ Because Taiwan is at the forefront in containing the transmission of COVID-19, as well as China’s expansionist ambition, a close bilateral US–Taiwan collaboration is natural. On 18 March 2020, the USA and Taiwan issued a joint statement to strengthen their consultation and cooperation in combatting COVID-19.^87^ This joint statement is of practical and political importance. Practically, it reflects the two sides’ wish to pursue closer collaboration; politically, after the USA switched its diplomatic relations from Taiwan to China, it marks the first time that the Taiwanese Minister of Foreign Affairs signed an agreement or issued a statement with a US authority, albeit still through the American Institute in Taiwan.^88^ In addition, the compelling need for cooperation in addressing the global COVID-19 crisis and for sharing experiences and information have similarly been felt by other countries.^89^ Leaders in Western countries have voiced their support for Taiwan’s participation in the WHO,^90^ signalling yet another wave of contestation against international public authority. Is multilateralism in freefall in the face of this global public health crisis?

## IV. Conclusion

The global COVID-19 crisis has driven governments worldwide to adopt exceptional measures to address imminent threats that may bend or break fundamental legal principles and constitutional guarantees in the process. While Taiwan has done an extraordinary job in response to this grand challenge, it is not immune to this dilemma. Drawing from the lessons of SARS and COVID-19, how do we reinvent the administrative state and ensure an adequate governance framework that safeguards core human rights and legal principles in cases of public health emergencies?

To begin, democratic governance and open society are cornerstones, not only for the state of normalcy, but also for the state of emergency. Congressional oversight and the principle of legal reservation remain the first gatekeepers and the foundation for safeguarding the rule of law and human rights. The COVID-19 Special Act, regardless of its controversial open-ended delegation, reflects this young democracy’s attempt to maintain its democratic legitimacy. With a large degree of transparency, the Chief of the CECC communicates daily with the affected population, which builds trust within the community and strengthens the legitimacy of its decisions. In response, human rights advocates and non-governmental organisations continue to point to the danger of human rights violations surrounding these emergency measures.

Furthermore, law, especially a set of pre-established procedures and substantive rules designed for risk regulation in times of such public health emergencies, is the second gatekeeper and remains the key instrument to guide and discipline Executive power. With its bitter lessons learned from SARS, Taiwan revised the CDC Act and relevant regulations, which provide a well-functioning framework to prepare for and respond to another public health emergency. This legal fabric helps prevent the Executive Branch from exercising its power in an arbitrary, capricious, misinformed or excessively disproportionate manner. A well-designed framework with pre-established procedures and substantive rules also enables effective *ex post* judicial oversight. Nevertheless, this second gatekeeper should go beyond a formalist understanding of legal safeguards and enlist innovative and dynamic approaches to broader socio-political checks and balances.

Finally, pandemics hit unevenly, both within this island country and across the globe. Emergency measures carry significant distributional justice implications, and the vulnerable are most susceptible to the threat of pandemics. Whereas Taiwan has so far produced a successful story in countering COVID-19, it has not been without cost: medical professionals are one example, and existing, chronic patients who are crowded out of treatment due to resource mobilisation are another. At the international level, the current health governance regime is by no means global or universal. Apart from Taiwan’s exclusion, it does not really reflect the interests of those most in need of assistance. Time will tell as COVID-19 hits the least-developed countries of the world. Therefore, a distributional justice logic deserves to be institutionalised as the normative anchor and the third gatekeeper of national and global health governance.

Taiwan’s experience in fighting COVID-19 presents an alternative model to China’s myth of authoritarian effectiveness. It also points to the failure of the current international public authority in moving beyond the political boundaries that diseases neither recognise nor respect. In the post-COVID-19 world, we deserve a new system of global health governance that delivers rather than undermines global public goods.

## Footnotes

1 “Taiwan National Infectious Disease Statistics System” (Centers for Disease Control), <http://nidss.cdc.gov.tw/en/SingleDisease.aspx?dc=1&dt=5&disease=19CoV> (last accessed 23 March 2020).

2 Communicable Disease Control Act 2019 (Taiwan) (CDC Act). The CDC Act was enacted in 1944 and was subsequently amended 15 times.

3 CDC Act, Art 14.

4 CDC Act, Art 9.

5 CDC Act, Art 17.

6 CDC Act, Art 44.

7 CDC Act, Arts 44 and 58–61.

8

G Lawson , “The Rise and Rise of the Administrative State” (1994) 107 Harvard Law Review 1231; HE McCurdy and DH Rosenbloom (eds), Revisiting Waldo’s Administrative State: Constancy and Change in Public Administration (Washington, DC, Georgetown University Press 2006 ); A Von Bogdandy etal (eds), *The Max Planck Handbooks in European Public Law Volume I: The Administrative State* (Oxford, Oxford University Press 2017). See also N Ma , “SARS and the Limits of the Hong Kong SAR Administrative State” (2004) 28(1) Asian Perspective 99 ; EA Posner and A Vermeule, “Crisis Governance in the Administrative State: 9/11 and the Financial Meltdown of 2008” (2008) <https://papers.ssrn.com/sol3/papers.cfm?abstract_id=1301164&fbclid=IwAR3t8R5gRc_P2vSqbVPGNgfMLM26zunzMm2tBMR6QfsdJfw26wAakhOw4yo> (last accessed 24 March 2020).

9 See, generally, H-Y Cheng etal, “Initial Rapid and Proactive Response for the COVID-19 Outbreak – Taiwan’s Experience” (2020) Journal of the Formosan Medical Association <https://doi.org/10.1016/j.jfma.2020.03.007> (last accessed 26 March 2020).

10 D Shapiro, “Taiwan Shows Its Mettle in Coronavirus Crisis, While the WHO is MIA” (*Brookings*, 19 March 2020) <https://www.brookings.edu/blog/order-from-chaos/2020/03/19/taiwan-shows-its-mettle-in-coronavirus-crisis-while-the-who-is-mia/> (last accessed 21 March 2020); G van der Wees, “Taiwan and the World Health Assembly” (*The Diplomat*, 10 May 2016) <https://thediplomat.com/2016/05/taiwan-and-the-world-health-assembly/> (last accessed 21 March 2020); H Tan, “Taipei Lashes Out at China for Blocking Taiwan’s Access to the World Health Organization” (*CNBC*, 6 February 2020) <https://www.cnbc.com/2020/02/06/coronavirus-taiwan-lashes-out-at-china-for-blocking-who-access.html> (last accessed 24 March 2020); “China’s Taiwan Policy Hurts World Health” (*WSJ*, 13 February 2020) <https://www.wsj.com/articles/chinas-taiwan-policy-hurts-world-health-11581618021> (last accessed 24 March 2020).

11 K-T Chen etal, “SARS in Taiwan: An Overview and Lessons Learned” (2005) 9(2) International Journal of Infectious Diseases 77; “Record SARS Toll in Taiwan After WHO Alert” (*The Irish Times*, 22 May 2003) <https://www.irishtimes.com/news/record-sars-toll-in-taiwan-after-who-alert-1.478506> (last accessed 21 March 2020); E Graham-Harrison, “Experience of SARS a Key Factor in Countries’ Response to Coronavirus” (*The Guardian*, 15 Mar 2020) <https://www.theguardian.com/world/2020/mar/15/experience-of-sars-key-factor-in-response-to-coronavirus> (last accessed 21 March 2020); G McGregor, “SARS Taught Taiwan How to Contain the Coronavirus Outbreak” (*Fortune*, 15 March 2020) <https://fortune.com/2020/03/15/coronavirus-taiwan-cases-response/> (last accessed 21 March 2020).

12 EL-L Chu, “The World Can Benefit From Taiwan’s WHO Participation” (*The Diplomat*, 11 February 2020) <https://thediplomat.com/2020/02/the-world-can-benefit-from-taiwans-who-participation/> (last accessed 21 March 2020); N Kassam, “As Wuhan Virus Spreads, Taiwan Has No Say at WHO” (*Foreign Policy*, 22 January 2020) <https://foreignpolicy.com/2020/01/22/china-health-coronavirus-wuhan-virus-spreads-taiwan-no-say-who/> (last accessed 21 March 2020); M Yun, “How Taiwan Is Containing Coronavirus – Despite Diplomatic Isolation by China” (*The Guardian*, 13 March 2020) <https://www.theguardian.com/world/2020/mar/13/how-taiwan-is-containing-coronavirus-despite-diplomatic-isolation-by-china> (last accessed 21 March 2020); B Blanchard, “Taiwan Says It Didn’t Need China’s Permission for WHO Meeting” (*Reuters*, 12 February 2020) <https://www.reuters.com/article/us-china-health-taiwan/taiwan-says-it-didnt-need-chinas-permission-for-who-meeting-idUSKBN2060JZ> (last accessed 24 March 2020).

13 Taiwan’s huge foreign reserve is also reflective of this “self-help” approach. J Kurlantzick, “Can the U.S. Emulate Asia’s Coronavirus Response?” (*CFR*, 18 March 2020) <https://www.cfr.org/in-brief/can-us-emulate-asias-coronavirus-response> (last accessed 23 March 2020); H Yip, “Fear of China Made Taiwan a Coronavirus Success Story” (*Foreign Policy*, 16 March 2020) <https://foreignpolicy.com/2020/03/16/taiwan-china-fear-coronavirus-success/> (last accessed 21 March 2020).

14 Graham-Harrison, supra, note 11; McGregor, supra, note 11.

15 Chen etal, supra, note 11, 82; AYM Chen , “The 2003 SARS Outbreaks in Taiwan” in Y Lu etal (eds), *Emerging Infections in Asia* (Berlin, Springer 2008).

16

Y Huang , “The SARS Epidemic and Its Aftermath in China: A Political Perspective” in S Knobler etal (eds), *Learning from SARS: Preparing for the Next Disease Outbreak* (Washington, DC, National Academies Press 2004).22553895

17 It should be noted that despite an observable convergence in adoption of the measure, a distinct contrast between Asian and Western countries can be observed regarding the pace of adoption: while Asian countries that closely experienced the SARS outbreak were generally quick to react (Hong Kong, Singapore, South Korea and Taiwan), Western countries have only adopted more restrictive measures more recently.

18 See, eg, D Calicchio, “Lockdowns, Stay-at-home Policies Multiply as Coronavirus Cases, Deaths Mount” (*Fox News*, 21 March 2020) <https://www.foxnews.com/health/lockdowns-stay-at-home-policies-multiply-as-coronavirus-cases-deaths-mount> (last accessed 21 March 2020).

19 See, eg, L Bicker, ‘Coronavirus in South Korea: How ‘Trace, Test and Treat’ May be Saving Lives’ (*BBC*, 12 March 2020) <https://www.bbc.com/news/world-asia-51836898> (last accessed 21 March 2020).

20 See, eg, K Vaswani, “Coronavirus: The Detectives Racing to Contain the Virus in Singapore” (*BBC*, 19 March 2020) <https://www.bbc.com/news/world-asia-51866102> (last accessed 21 March 2020); H Cheung, “Coronavirus: What Could the West Learn from Asia?” (*BBC*, 21 March 2020) <https://www.bbc.com/news/world-asia-51970379> (last accessed 21 March 2020).

21 See, eg, “Coronavirus: Australia to Close Pubs, Cafes and Places of Worship” (*BBC*, 22 March 2020) <https://www.bbc.com/news/world-australia-51992357> (last accessed 22 March 2020).

22 See, eg, Cheung, supra, note 20; “Coronavirus: Travel Restrictions, Border Shutdowns by Country” (*Al-Jazeera* 22 March 2020) <https://www.aljazeera.com/news/2020/03/coronavirus-travel-restrictions-border-shutdowns-country-200318091505922.html> (last accessed 22 March 2020).

23 Emergencies, such as disease outbreaks, natural disasters or terrorist attacks, often call for considerable discretion conferred to the administrative agencies, resulting in “the hour of the executive” for a legal system. See FB Bastos and A de Ruijter , “Break or Bend in Case of Emergency?: Rule of Law and State of Emergency in European Public Health Administration” (2019) 10(4) European Journal of Risk Regulation 610 , 610.

24
ibid, pp 611–12. See also D Curtin , “Challenging Executive Dominance in European Democracy” (2014) 77(1) Modern Law Review 1 ; SG Knowles , “Learning from Disaster?: The History of Technology and the Future of Disaster Research” (2014) 55(4) Technology and Culture 773 ; A Vermeule , Law’s Abnegation: From Law’s Empire to the Administrative State (Cambridge, MA, Harvard University Press 2016 ).

25 For instance, while Taiwan’s Constitutional Court upheld all of the necessary measures adopted by the government to counter the SARS crisis, the Court also warned that the lack of a reasonable maximum time for compulsory quarantine and the right to appeal should be reassessed and amended under the broader legal framework for communicable disease control. See W-C Chang, “Taiwan’s Fight against COVID-19: Constitutionalism, Laws, and the Global Pandemic” (*Verfassungsblog on Matters Constitutional*, 21 March 2020) <https://verfassungsblog.de/taiwans-fight-against-covid-19-constitutionalism-laws-and-the-global-pandemic/> (last accessed 21 March 2020); see also Taiwan Constitutional Court J.Y. Interpretation No. 690 (30 September 2011) (Interpretation No. 690); Taiwan Centers for Disease Control, “Prevention Strategies against Emerging Infectious Disease in Taiwan under Globalization” (2020) 40 Angle Health Law Review 7.

26

S Hameiri and L Jones , Governing Borderless Threats (Cambridge, Cambridge University Press 2015) pp 25–26 .

27 A Neve, “Human Rights Muse Remain at the Heart of Our Response to Coronavirus Crisis” (*The Star*, 19 March 2020) <https://www.thestar.com/opinion/contributors/2020/03/19/human-rights-must-remain-at-the-very-heart-of-our-response-to-the-coronavirus-crisis.html> (last accessed 26 March 2020).

28 See V Rollet , “Framing SARS and H5N1 as an Issue of National Security in Taiwan: Process, Motivations and Consequences” (2014) 37 Extrême-Orient 141 , 144; DP Fidler , “To Declare or Not to Declare: The Controversy over Declaring a Public Health Emergency of International Concern for the Ebola Outbreak in the Democratic Republic of the Congo” (2019) 14(2) Asian Journal of WTO & International Health Law and Policy 287 , 320–23.

29

S-L Huang , M Bernard and H-H Huang , “Mistakes to Avoid in Order to Stop the Outbreak of A New Global Virus: The Case of SARS in Taiwan” (2004) 45 Hungkuang Law Review 159 , 166.

30

J Schwartz and M-Y Yen , “Toward a Collaborative Model of Pandemic Preparedness and Response: Taiwan’s Changing approach to Pandemics” (2017) 50(2) Journal of Microbiology, Immunology and Infection 125 10.1016/j.jmii.2016.08.010PMC710504028089098, 127–28.

31 See Yip, supra, note 13.

32 See Y Lee and B Blanchard, “Taiwan Says WHO Ignored Its Coronavirus Questions at Start of Outbreak” (*US News*, 24 March 2020) <https://www.usnews.com/news/world/articles/2020-03-24/taiwan-says-who-ignored-its-coronavirus-questions-at-start-of-outbreak> (last accessed 24 March 2020); “Taiwan Says WHO Failed to Act on Coronavirus Transmission Warning” (*Financial Times*, 20 March 2020) <https://www.usnews.com/news/world/articles/2020-03-24/taiwan-says-who-ignored-its-coronavirus-questions-at-start-of-outbreak> (last accessed 26 March 2020).

33 CJ Wang etal, “Response to COVID-19 in Taiwan: Big Data Analytics, New Technology, and Proactive Testing” (2020) JAMA <https://www.ft.com/content/2a70a02a-644a-11ea-a6cd-df28cc3c6a68> (last accessed 24 March 2020).

34 CDC (Taiwan), “In Response to Pneumonia Outbreak in Wuhan, China, Taiwan CDC Advises Travelers Visiting Outbreak Area to Take Relevant Precautions Throughout Trip and After Returning to Taiwan” (*CDC*, 6 January 2020) <https://www.cdc.gov.tw/En/Bulletin/Detail/Dbg1J9leIDoxseqTHMiaxQ?typeid=158> (last accessed 26 March 2020); CDC (Taiwan), “In Response to Pneumonia Outbreak in Wuhan, China and Related Test Results, Taiwan CDC Remains in Touch with China and World Health Organization and Taiwan Maintains Existing Disease Control and Prevention Efforts” (*CDC*, 9 January 2020) <https://www.cdc.gov.tw/En/Bulletin/Detail/4N96uF-2yK-d7dEFFcwa0Q?typeid=158> (last accessed 26 March 2020).

35 CDC (Taiwan), “Two Experts from Taiwan Visit Wuhan to Understand and Obtain Information on Severe Special Infectious Pneumonia Outbreak; Taiwan CDC Raises Travel Notice Level for Wuhan to Level 2” (*CDC*, 20 January 2020) <https://www.cdc.gov.tw/En/Bulletin/Detail/4N96uF-2yK-d7dEFFcwa0Q?typeid=158> (last accessed 26 March 2020).

36 CDC Act, Art 3.

37 CDC Act, Art 17.

38 See Wang etal, supra, note 33, Supplementary Online Content.

39 See Yip, supra, note 13.

40 See CR Sunstein , Laws of Fear: Beyond the Precautionary Principle (Cambridge, Cambridge University Press 2005) pp 13–15 ; see also MBA van Asselt and E Vos , “The Precautionary Principle and the Uncertainty Paradox” (2006) 9(4) Journal of Risk Research 313 , 314–18. It should be noted that Sunstein argues that for the global COVID-19 crisis, for which “it’s unusually challenging to engage in quantitative cost–benefit analysis”, a zero-risk mentality makes no sense. While the cost–benefit analysis usually points out that it is dangerous to be overcautious, “[t]he coronavirus is different”. In the face of the COVID-19 crisis, Sunstein even argues that cost–benefit analysis should support very aggressive precautions. CR Sunstein, “This Time the Numbers Show We Can’t Be Too Careful” (*Bloomberg*, 27 March 2020) <https://www.bloomberg.com/opinion/articles/2020-03-26/coronavirus-lockdowns-look-smart-under-cost-benefit-scrutiny> (last accessed 27 March 2020).

41 V Pu, “The Coronavirus Outbreak: How Democratic Taiwan Outperformed Authoritarian China” (*The Diplomat,* 27 February 2020) <https://thediplomat.com/2020/02/the-coronavirus-outbreak-how-democratic-taiwan-outperformed-authoritarian-china> (last accessed 24 March 2020); J Lanier and EG Weyl, “How Civic Technology Can Help Stop a Pandemic” (*Foreign Affairs*, 20 March 2020) <https://thediplomat.com/2020/02/the-coronavirus-outbreak-how-democratic-taiwan-outperformed-authoritarian-china> (last accessed 26 March 2020).

42 S Lin, “Virus Outbreak: Supplies of Food, Daily Necessities Sufficient” (*Taipei Times*, 21 March 2020) <https://www.taipeitimes.com/News/taiwan/archives/2020/03/21/2003733109> (last accessed 26 March 2020).

43 In order to take charge of disinformation dissemination during epidemics, not only the CDC Act Arts 9 and 63 but also the Social Order Maintenance Act (SOM Act) Article 63(I)(5) can be cited as a legal basis to penalise those who spread fake news with imprisonment or a fine or the mass media companies who refuse to help correct disinformation upon notification by the authority.

44 B Hioe and L Wooster, “Taiwan News Publishes COVID-19 Misinformation as Epidemic Spreads” (*New Bloom*, 12 February 2020) <https://newbloommag.net/2020/02/12/coronavirus-taiwan-news> (last accessed 23 March 2020).

45 See, eg, M-H Huang , “Fake News, Social Media, and the Freedom of Speech in the Internet Era (in Chinese)” (2019) 292 Taiwan Law Review 5 ; Taiwan Association for Human Rights, “Guanjhih Bushih Syunsi, Ying Jiangu Yanlunzihyou (管制不實訊息，應兼顧言論自由) [Fake News Regulations Should Consider the Protection of the Freedom of Expression]” (*TAHR*, 13 January 2020) <https://newbloommag.net/2020/02/12/coronavirus-taiwan-news> (last accessed 24 March 2020).

46 But these regulations require a delicate balance because excessive controls of freedom of expression and freedom of the press would also present a threat to democracy, as much as would fake news.

47 Taiwan’s risk regulation against the global COVID-19 crisis has so far been one of the successful stories of the crisis; ibid. In addition, among others, Korea is also taking a proactive approach to combating COVID-19. See M Fisher and S-H Choe, “How South Korea Flattened the Curve” (*NY Times*, 23 March 2020) <https://www.nytimes.com/2020/03/23/world/asia/coronavirus-south-korea-flatten-curve.html?_ga=2.194021805.1487284911.1585243014-1702614851.1585243014> (last accessed 26 March 2020).

48 At that time, four million medical masks were released to the market daily, up to three medical masks were allowed per purchase at convenience stores, local pharmacies and medical supply stores and the price was fixed at US$0.27 apiece. On 3 February, citizens could purchase up to two medical masks per week with their NHI card at the price of US$0.17 apiece. During the same period, Taiwan’s High Prosecutors Office launched a national campaign to stop profiteering by raising prices on critical medical supplies. Wang etal, supra, note 33, Supplementary Online Content; K Chuan etal, “Taiwan Government Extends Requisitioning of Masks, Ban on Exports” (*Focus Taiwan*, 13 February 2020) <https://focustaiwan.tw/society/202002130012> (last accessed 24 March 2020).

49 P-J Teng, “Taiwan Sets up 60 Face Mask Production Lines in a Month” (*Taiwan News*, 6 March 2020) <https://www.taiwannews.com.tw/en/news/3891193> (last accessed 24 March 2020). This was a government project to establish 60 production lines in a month by working with 15 local manufacturers and relevant business associations, which even helped accomplish the project ahead of schedule.

50 K Wei, “Coronavirus: How Taiwan is Cranking up Mask Production to Meet Shortfall” (*Straits Times*, 3 March 2020) <https://www.straitstimes.com/asia/east-asia/coronavirus-how-taiwan-is-cranking-up-mask-production-to-meet-shortfall> (last accessed 24 March 2020). The government plans to spend another US$4.18 million to add 30 more production lines, which will be established in local factories by the end of March.

51 “U.S.-Taiwan Joint Statement” (*AIT*, 18 March 2020) <https://www.ait.org.tw/u-s-taiwan-joint-statement> (last accessed 26 March 2020); J Gehrke, “Taiwan Plans to Donate 100,000 Hospital Masks to US per Week” (*Washington Examiner*, 9 March 2020) <https://www.washingtonexaminer.com/policy/foreign/taiwan-plans-to-donate-100-000-hospital-masks-to-us-per-week> (last accessed 26 March 2020).

52 CDC Act, Art 14; H-Y Kao etal, “Taiwan’s Experience in Hospital Preparedness and Response for Emerging Infectious Diseases” (2017) 15(2) Health Security 175.2841874510.1089/hs.2016.0105PMC5404255

53 S Hsiao, “Virus Outbreak: Groups Calls for Protection of Front-Line Medics” (*Taipei Times*, 12 February 2020) <http://www.taipeitimes.com/News/taiwan/archives/2020/02/12/2003730832> (last accessed 26 March 2020).

54 “Kuai Sinwun/Jiating Zeren Buneng Fang! Chen Shihjhong: Liousyuehsheng Wujiakeguei Tsai Kaolyu Jijhong Jianyi (快新聞／家庭責任不能放！陳時中：留學生無家可歸才考慮集中檢疫) [Just In: Home Responsibility Can’t Be Neglected! Chen Si-Chung: Oversea Students Will Only Be Isolated and Tested if They Have No Place to Go]” (*FTV*, 18 March 2020) <https://bit.ly/2UFUW6d> (last accessed 26 March 2020).

55 There has been no research done regarding whether this centralised system and prioritisation methodology would unfairly affect other healthcare priorities (such as those for non-communicable diseases). B Jennings and J Arras, “Ethical Guidance for Public Emergency Preparedness and Response: Highlighting Ethics and Values in Vital Public Health Service” (2008) <https://www.cdc.gov/od/science/integrity/phethics/docs/white_paper_final_for_website_2012_4_6_12_final_for_web_508_compliant.pdf> (last accessed 26 March 2020).

56 Due to the significant uncertainty of a novel virus, it is important to confer more discretion to physicians so that they can adopt timely and effective measures. SA Raza, “What ‘Distributive Justice’ Means for Doctors Treating COVID-19” (*3 Quarks Daily*, 22 March 2020) <https://www.3quarksdaily.com/3quarksdaily/2020/03/what-distributive-justice-means-for-doctors-treating-covid-19.html> (last accessed 26 March 2020).

57 For example, the segments of the population upon whom the burden is likely to fall and the impacts of this should be evaluated, and cost–effectiveness analysis should not be the only evaluation tool. S Hoffman , “Preparing for Disaster: Protecting the Most Vulnerable in Emergencies” (2008) 42 UC Davis Law Review 42 .

58 Jennings and Arras, supra, note 55. DE Vawter etal, “For the Good of Us All: Ethically Rationing Health Resources in Minnesota in A Severe Influenza Pandemic” (2010) <https://www.health.state.mn.us/communities/ep/surge/crisis/ethics.pdf> (last accessed 26 March 2020).

59 Bastos and de Ruijter, supra, note 23. See also EJ Emanuel, “Fair Allocation of Scarce Medical Resources in the Time of Covid-19” (2020) New England Journal of Medicine <https://www.nejm.org/doi/pdf/10.1056/NEJMsb2005114> (last accessed 26 March 2020).

60 Wang etal, supra, note 33, Supplementary Online Content.

61 N Aspinwall, “Taiwan Closes Borders in Preparation for Possible ‘Second Wave’ of the Coronavirus” (*The Diplomat*, 20 March 2020) <https://thediplomat.com/2020/03/taiwan-closes-borders-in-preparation-for-possible-second-wave-of-the-coronavirus/> (last accessed 26 March 2020).

62 W Chen and Y Chiang, “Taiwan Amends Travel Ban on Medical Personnel” (*Focus Taiwan*, 24 February 2020) <https://focustaiwan.tw/society/202002240006> (last accessed 26 March 2020). These border control measures find their legal basis in quarantine regulations, which have been substantially revised as a result of the critique of the Constitutional Court following the SARS crisis. Y-C Su, “When Ethical Reform Became Law: The Constitutional Concerns Raised by Recent Legislation in Taiwan” (2014) 40(7) Journal of Medical Ethics 484. See also Interpretation No. 690, supra, note 25.

63 A poll taken in February 2020 indicated high approval ratings for the government’s response to the crisis. S Beale, “Taiwan’s Containment of COVID-19 Outbreak Demonstrates Importance of Rapid Response, Including Fast Access to Clinical Laboratory Tests” (*Dark Daily*, 23 March 2020) <https://www.darkdaily.com/taiwans-containment-of-covid-19-outbreak-demonstrates-importance-of-rapid-response-including-fast-access-to-clinical-laboratory-tests> (last accessed 26 March 2020).

64 See Chang, supra, note 25.

65 I-C Lee, “Virus Outbreak: Level 3 Alert Issued for More Nations” (*Taipei Times*, 17 March 2020) <https://www.taipeitimes.com/News/front/archives/2020/03/17/2003732835> (last accessed 24 March 2020).

66 Even though Article 24 of the Physician Act required physicians to follow the authority’s orders in cases of public health emergencies, it is debatable whether physicians’ obligations include surrendering their liberty to travel abroad in their personal life.

67 C-C Chen etal, “COVID-19 Patient Frequently Used Public Transport in Greater Taipei” (*Focus Taiwan*, 27 February 2020) <https://focustaiwan.tw/society/202002270018> (last accessed 22 March 2020).

68 C-T Cheng, “Taiwan Implements Electronic Security System to Track Individuals under Quarantine” (*Taiwan News*, 18 March 2020) <https://www.taiwannews.com.tw/en/news/3899718> (last accessed 22 March 2020).

69 CO Buckee, “Aggregated Mobility Data Could Help COVID-19” (2020) <https://science.sciencemag.org/content/early/2020/03/20/science.abb8021/tab-pdf> (last accessed 26 March 2020).

70

C-H Chang , “Eyes on the Road Program in Taiwan – Information Privacy Issues under the Taiwan Personal Data Protection Act” (2014) 31 John Marshall Journal of Information Technology & Privacy Law 145 ; S Peng , “Privacy and the Construction of Legal Meaning in Taiwan” (2003) 37 International Lawyer 1037 .

71 CDC Act, Art 58(I)(4).

72 Special Act for Prevention, Relief and Revitalization Measures for Severe Pneumonia with Novel Pathogens 2020 (Taiwan) (COVID-19 Special Act), Art 8.

73 Unfortunately, the government has yet to specify different levels of privacy for different users, which can alleviate privacy invasion when personal data are widely shared without consent in a crisis. See generally B Milano, “Restricting Civil Liberties Amid the COVID-19 Pandemic” (*Harvard Law Today*, 21 March 2020) <https://today.law.harvard.edu/restricting-civil-liberties-amid-the-covid-19-pandemic/?utm_source=hltFacebook&fbclid=IwAR3amjujASJmKQEpVbEUQHBhz7zP2aEAMCtc2fp1lFnExDY426N1K_RhzZc> (last accessed 26 March 2020).

74 Taiwan Association for Human Rights, “Protecting Civil Liberties During a Public Health Crisis” (*Medium*, 19 March 2020) <https://medium.com/@tahr1984/protecting-civil-liberties-during-a-public-health-crisis-1de3c6d8e724> (last accessed 24 March 2020). It seems unnecessary and disproportionate to maintain years of travel history in the NHI ID card since COVID-19 has only a two-week incubation period. Furthermore, the lack of transparency when processing personal data also unfairly affects the substance and detail of the legal challenges individuals could have made to these privacy invasive measures. Thus, detailed information regarding how personal data are collected, the retention period for the data, the tools used to process the data and how the data analysis guides public health decisions should be published and made available to the public. Moreover, these measures failed to provide individuals fair opportunity to challenge in a timely manner the limits imposed on them based upon the analysis of personal data. Last but not least, there is no withdrawal mechanism either, which would set out the conditions for when the government should roll back the invasive programmes created in the name of public health. This mechanism is important because the data surveillance measures might long outlive the COVID-19 crisis.

75 N Singer and S Choe, “As Coronavirus Surveillance Escalates, Personal Privacy Plummets” (*NY Times*, 23 March 2020) <https://www.nytimes.com/2020/03/23/technology/coronavirus-surveillance-tracking-privacy.html?_ga=2.228650268.1487284911.1585243014-1702614851.1585243014> (last accessed 26 March 2020).

76

O Gross , “Extra-Legality and the Ethic of Political Responsibility” in VV Ramraj (ed), Emergencies and the Limits of Legality (Cambridge, Cambridge University Press 2008) p 240 .

77

L Bengtsson and M Rhinard , “Securitisation across Borders: The Case of ‘Health Security’ Cooperation in the European Union” (2019) 42 West European Politics 346 . To be sure, human rights are oftentimes limited or even violated on the grounds of protecting public health in times of emergency. However, “a global health crisis should not be used to normalize oppressive surveillance measures, which do not comply with international human rights standards”. ML Stasi and B Bukovska, “COVID-19 Surveillance Must Not Be Used as an Excuse to Entrench Surveillance” (*Just Security*, 20 March 2020) <https://www.justsecurity.org/69282/covid-19-surveillance-must-not-be-used-as-an-excuse-to-entrench-surveillance/?fbclid=IwAR3kb3qvB2Sg6zeXzxAGCQU3tscfirK-XmN850dF4qtmWHrXS67e5JhaRn8> (last accessed 21 March 2020).

78

B Ackerman , “The Emergency Constitution” (2004) 113 Yale Law Journal 1029 , 1057.

79 Lawrence Gostin and Emily Mok identify six grand challenges in global health governance, which include accountability and transparency arising from international policy-making. LO Gostin and EA Mok , “Grand Challenges in Global Health Governance” (2009) 90 British Medical Bulletin 7 1937680210.1093/bmb/ldp014, 15–16.

80 See, eg, D Dyzenhaus , The Constitution of Law: Legality in a Time of Emergency (Cambridge, Cambridge University Press 2006 ) ch 3.

81 Constitution (Taiwan), Art 43. In case of a natural calamity, an epidemic or a national financial or economic crisis that calls for emergency measures, the President, during the recess of the Legislative Yuan, may, by resolution of the Executive Yuan Council, and in accordance with the Law on Emergency Decrees, issue emergency decrees, proclaiming such measures as may be necessary to cope with the situation.

82 S Wu (吳書緯), “Shih Li Ti Sioufa Bimian Fangyi Tebieh Tiaoli Kongbai Shouchyua (時力提修法 避免防疫特別條例空白授權) [NPP Proposes Amendments to Prevent Blank Authorization]” (*LTN*, 24 March 2020) <https://news.ltn.com.tw/news/politics/breakingnews/3110511> (last accessed 24 March 2020).

83 More generally on the role played by civil society in Taiwan during this COVID-19 pandemic, see E Chao, “Robust Civil Society Key to Success” (*Taipei Times*, 25 March 2020) <https://www.taipeitimes.com/News/editorials/archives/2020/03/25/2003733313?fbclid=IwAR1PeNLCh-abQ0Gm8vWXKD9e8hNncFgtDkoh5etsoDMSKeAFl3rEqqyS2Sw> (last accessed 26 March 2020).

84 Taiwan Association for Human Rights, “Dang Fajhih Guo Yushang Bingdu: Wu Lanyong Gaigua Tiaokuan, Fangyi Yu Minjhu Tsaineng Gongtsun (當法治國遇上病毒：勿濫用概括條款，防疫與民主才能共存) [When Rule of Law Meets Virus: Don’t Abuse the General Clause so That Epidemic Prevention Can Co-exist with Democracy]” (*TAHR*, 18 March 2020) <https://www.tahr.org.tw/news/2622> (last accessed 24 March 2020).

85 See generally A von Bogdandy and PA Villarreal, “International Law on Pandemic Response: A First Stocktaking in Light of the Coronavirus Crisis” (2020) Max Planck Institute for Comparative Public Law & International Law Research Paper Series 7/2020 <https://papers.ssrn.com/sol3/papers.cfm?abstract_id=3561650> (last accessed 27 March 2020).

86 KM Campbell and R Doshi, “The Coronavirus Could Reshape Global Order: China Is Maneuvering for International Leadership as the United States Falters” (*Foreign Affairs*, 18 March 2020) <https://www.foreignaffairs.com/articles/china/2020-03-18/coronavirus-could-reshape-global-order> (last accessed 26 March 2020). On a related note, China has even been exploiting this COVID-19 outbreak to undermine democratic systems and push its propaganda against the USA. See P Rough, “How China Is Exploiting the Coronavirus to Weaken Democracies” (*Foreign Policy*, 25 March 2020) <https://foreignpolicy.com/2020/03/25/china-coronavirus-propaganda-weakens-western-democracies/?fbclid=IwAR0OHtEP6swVob_aZIcl0O3RA4OqXxllkw2o_2EQx8nP7USCnnK6tQZLX1E> (last accessed 27 March 2020).

87 US–Taiwan Joint Statement, supra, note 51.

88
ibid.

89 For instance, the European Commission, through its Economic and Trade Office in Taiwan, co-sponsored collaboration with the chief research institution of Taiwan, Academia Sinica, with a view to earlier control and containment of this pandemic through joint research. F Grzegorzewki, “#EU and #Taiwan Academia Sinica working together on the most advanced rapid tests and vaccines for #COVID. With 15-minutes reliable tests, we will defeat” <https://twitter.com/grzegorzewskif/status/1240212023394422784> (last accessed 21 March 2020).

90 “Taiwan: EU Supports Taipei’s Bid to Join WHO” (*Stratfor*, 31 January 2020) <https://worldview.stratfor.com/situation-report/taiwan-eu-supports-taipeis-bid-join-who> (last accessed 26 March 2020); B Blanchard and YL Taiwan, “U.S. Increases Support for Taiwan, China Threatens to Strike Back” (*Reuters*, 27 March 2020) <https://www.reuters.com/article/us-taiwan-usa/u-s-increases-support-for-taiwan-in-recognition-battle-with-china-idUSKBN21E0B7> (last accessed 27 March 2020); “Japan PM Abe Calls for Taiwan’s Participation in WHO as Coronavirus Spreads” (*Kyodo News*, 30 January 2020) <https://english.kyodonews.net/news/2020/01/cff2af87f289-abe-calls-for-taiwans-participation-in-who-as-coronavirus-spreads.html> (last accessed 26 March 2020); CW Nelson, “COVID-19: Time for WHO to Reconsider Its Stance Towards Taiwan’ (2020) 579 Nature 193.

